# Risk factors analysis of lateral cervical lymph node metastasis in papillary thyroid carcinoma: a retrospective study of 830 patients

**DOI:** 10.1186/s12957-024-03455-w

**Published:** 2024-06-21

**Authors:** Haifeng Zhong, Qingxin Zeng, Xi Long, Yeqian Lai, Jiwei Chen, Yuedong Wang

**Affiliations:** 1 Department of Thyroid Surgery, Meizhou People’s Hospital, Meizhou Academy of Medical Sciences, Add: No. 63 Huangtang Road, Meijiang District, Meizhou, China; 2 Department of Radiology, Meizhou People’s Hospital, Meizhou Academy of Medical Sciences, Meizhou, China

**Keywords:** Papillary thyroid carcinoma, Lateral cervical lymph node metastasis, Central lymph node metastasis, Lymph node metastasis

## Abstract

**Objective:**

The aim of this study is to investigate the risk factors for lateral cervical lymph node metastasis in papillary thyroid carcinoma (PTC).

**Methods:**

Clinicopathological data (age, gender, Hashimoto’s thyroiditis, preoperative circulating tumor cells (CTCs), multifocal, maximum lesion diameter, invaded capsule, T stage, and lymph node metastasis) of 830 PTC patients diagnosed and treated in Meizhou People’s Hospital from June 2021 to April 2023 were collected. The related factors of lateral cervical lymph node metastasis were analyzed.

**Results:**

There were 334 (40.2%), and 103 (12.4%) PTC patients with central lymph node metastasis, and lateral cervical lymph node metastasis, respectively. Compared with patients without lateral cervical lymph node metastasis, PTC patients with lateral cervical lymph node metastasis had a higher proportion of multifocal, maximum lesion diameter > 1 cm, invaded capsule, T3-T4 stage. Regression logistic analysis showed that male (odds ratio (OR): 2.196, 95% confidence interval (CI): 1.279–3.769, *p* = 0.004), age < 55 years old (OR: 2.057, 95% CI: 1.062–3.988, *p* = 0.033), multifocal (OR: 2.759, 95% CI: 1.708–4.458, *p* < 0.001), maximum lesion diameter > 1 cm (OR: 5.408, 95% CI: 3.233–9.046, *p* < 0.001), T3-T4 stage (OR: 2.396, 95% CI: 1.241–4.626, *p* = 0.009), and invaded capsule (OR: 2.051, 95% CI: 1.208–3.480, *p* = 0.008) were associated with lateral cervical lymph node metastasis.

**Conclusions:**

Male, age < 55 years old, multifocal, maximum lesion diameter > 1 cm, T3-T4 stage, and invaded capsule were independent risk factors for lateral cervical lymph node metastasis in PTC.

## Introduction

Thyroid cancer is one of the malignant tumors of endocrine system [1]. In recent years, the global incidence of thyroid cancer has increased year by year [2]. Thyroid cancer is the most common endocrine tumor in the world, with age-standardized incidence rate increasing over time [3]. In recent years, the increase in overall cancer rates among adolescents and young adults has been driven primarily by thyroid cancer [4]. The incidence of thyroid cancer in China is expected to increase significantly in the next 20 years [5]. Thyroid cancer is divided into four main types, including papillary thyroid carcinoma (PTC), follicular thyroid carcinoma (FTC), medullarly thyroid carcinoma (MTC), and anaplastic thyroid carcinoma (ATC) [6, 7].

PTC originates from thyroid follicular epithelial cells and is the most common histopathological type of thyroid cancer [8]. Some studies have found 20-80% of PTC patients had lymph node metastasis [9–11]. The presence of lymph node metastasis not only affects the prognosis of patients, but also increases the postoperative recurrence rate and mortality [12]. Cervical lymph node metastasis of PTC usually presents as a stepped metastasis, first to the central cervical lymph node and then to the lateral cervical lymph node, which is the second station of cervical lymph node metastasis in PTC patients [13]. In addition to thyroid surgery, the surgical treatment of PTC patients also includes lymph node management, which is divided into central lymph node dissection and lateral cervical lymph node dissection [14, 15]. At present, the prophylactic lateral neck dissection has a certain role in the treatment of patients with PTC [16, 17]. Therefore, understanding the risk factors of lateral cervical lymph node metastasis in PTC patients has important clinical significance for determining the scope of lateral lymph node dissection and its indication.

Lateral cervical lymph node metastasis of PTC is common, and there are many risk factors, including age [18], male [19, 20], tumor size [21], extrathyroidal extension [20], multifocal [22, 23], and other clinicopathological features. However, there are also some studies showed that age [20], gender [24], and multifocal [25] have no correlation with lateral cervical lymph node metastasis of PTC. Song RY et al. found that minimal extrathyroidal extension is associated with lymph node metastasis of PTC, but not gross extrathyroidal extension [26]. The differences in the results of the above studies may be due to the differences in the study cohorts of different studies and the selection of clinical data included. This study evaluated the relationship between the clinicopathological features and lateral cervical lymph node metastasis of PTC. The purpose of this study was to provide reference data for the risk prediction of lateral cervical lymph node metastasis in patients with PTC.

## Materials and methods

### Subjects

The study included 830 PTC patients who were hospitalized in Meizhou People’s Hospital, from June 2021 to April 2023. Inclusion criteria: (1) All PTC patients were confirmed by histopathology and imaging examination; (2) Complete records of medical records received in our hospital for diagnosis and treatment; (3) No history of head and neck radiation. Exclusion criteria: (1) Previous history of other malignant tumor diseases; (2) Pathological types other than papillary thyroid carcinoma; (3) Patients with dysfunction of important organs. This study was supported by the Ethics Committee of the Meizhou People’s Hospital.

### Data collection

Clinicopathological features of the patients were retrospectively collected from the medical records system of Meizhou People’s Hospital, including age, gender, Hashimoto’s thyroiditis, preoperative circulating tumor cells (CTCs), multifocal, maximum lesion diameter, invaded capsule, T stage, and lymph node metastasis.

3 ml of peripheral blood were withdrawn into an EDTA-containing anticoagulant vacuum tube from each patient one day before surgery. CTCs were enriched and quantifed using the CytoploRare Kit according to product manuals (Genosaber Biotech, Shanghai, China). “FU/3mL” was a self-designated CTC unit which derived from a standard curve was used to denote the amount of folate receptor-positive CTCs (FR^+^ CTCs) in 3 ml peripheral blood.

### Statistical analysis

SPSS statistical software version 26.0 (IBM Inc., USA) was used for data analysis. Association between central lymph node metastasis, lateral cervical lymph node metastasis and the clinicopathological features of PTC patients was evaluated by *Chi*-square test or Fisher’s exact test. Univariate analysis and multivariate regression logistic analysis were used to evaluate the relationship between the clinicopathological features and central lymph node metastasis, lateral cervical lymph node metastasis in PTC patients, respectively. *p* < 0.05 was set as statistically significant.

## Results

### Clinicopathological features of PTC patients

A total of 830 patients with PTC were assessed in the study. There were 152 (18.3%) PTC patients were men and 678 (81.7%) were women; there were 652 (78.6%) PTC patients were younger than 55 years old and 178 (21.4%) PTC patients were ≥ 55 years old. These results suggest that PTC patients are mainly young women. There were 213 (25.7%) PTC patients combined with Hashimoto’s thyroiditis. There were 311 (37.5%) PTC patients with preoperative CTCs < 8.7 FU/3mL, and 519 (62.5%) PTC patients with preoperative CTCs ≥ 8.7 FU/3mL. There were 231 (27.8%) cases, 249 (30.0%) cases, and 348 (41.9%) cases with multifocal, maximum lesion diameter > 1 cm, and invaded capsule, respectively. There were 764 (92.0%) and 66 (8.0%) PTC patients in T1-T2 and T3-T4 stages, respectively. There were 334 (40.2%), and 103 (12.4%) PTC patients with central lymph node metastasis, and lateral cervical lymph node metastasis, respectively (Table 1).

**Table 1:** The clinicopathological features of PTC patients

Clinicopathological features	PTC patients (*n* = 830)
Gender	
Male, n (%)	152 (18.3%)
Female, n (%)	678 (81.7%)
Age (Years)	
< 55, n (%)	652 (78.6%)
≥ 55, n (%)	178 (21.4%)
Hashimoto’s thyroiditis	
No, n (%)	617 (74.3%)
Yes, n (%)	213 (25.7%)
Preoperative circulating tumor cells (CTCs) (FU/3mL)	
< 8.7	311 (37.5%)
≥ 8.7	519 (62.5%)
Multifocal	
No, n (%)	599 (72.2%)
Yes, n (%)	231 (27.8%)
Maximum lesion diameter	
≤ 1 cm, n (%)	581 (70.0%)
> 1 cm, n (%)	249 (30.0%)
Invaded capsule	
No, n (%)	482 (58.1%)
Yes, n (%)	348 (41.9%)
T stage	
T1-T2, n (%)	764 (92.0%)
T3-T4, n (%)	66 (8.0%)
Central lymph node metastasis	
No, n (%)	496 (59.8%)
Yes, n (%)	334 (40.2%)
Lateral cervical lymph node metastasis	
No, n (%)	727 (87.6%)
Yes, n (%)	103 (12.4%)

PTC: papillary thyroid carcinoma; CTC: circulating tumor cell; FU: folate receptor unit

### Comparison of clinicopathological features among PTC patients with or without central lymph node metastasis

There were 496 (496/830, 59.8%) PTC patients without and 334 (334/830, 40.2%) PTC patients with central lymph node metastasis. Compared with patients without central lymph node metastasis, PTC patients with central lymph node metastasis had a higher proportion of male patients (23.4% vs. 14.9%) (*p* = 0.002), and patients with < 55 years of age (83.5% vs. 75.2%) (*p =* 0.004). The proportion of multifocal (38.9% vs. 20.4%) (*p* < 0.001), maximum lesion diameter > 1 cm (48.2% vs. 17.7%) (*p* < 0.001), invaded capsule (56.0% vs. 32.5%) (*p* < 0.001), and T3-T4 stage (13.5% vs. 4.2%) (*p* < 0.001) in PTC patients with central lymph node metastasis was higher than that in PTC patients without central lymph node metastasis, respectively. There were no statistically significant differences in proportion of Hashimoto’s thyroiditis, and preoperative CTCs between the two groups (Table 2).

**Table 2:** Comparison of clinicopathological features among PTC patients with or without central lymph node metastasis

Clinicopathological features	Central lymph node metastasis		*p* values
	No (*n* = 496)	Yes (*n* = 334)	
Gender			
Male, n (%)	74(14.9%)	78(23.4%)	0.002
Female, n (%)	422(85.1%)	256(76.6%)	
Age (Years)			
< 55, n (%)	373(75.2%)	279(83.5%)	0.004
≥ 55, n (%)	123(24.8%)	55(16.5%)	
Hashimoto’s thyroiditis			
No, n (%)	373(75.2%)	244(73.1%)	0.517
Yes, n (%)	123(24.8%)	90(26.9%)	
Preoperative circulating tumor cells (CTCs) (FU/3mL)			
< 8.7	185(37.3%)	126(37.7%)	0.942
≥ 8.7	311(62.7%)	208(62.3%)	
Multifocal			
No, n (%)	395(79.6%)	204(61.1%)	< 0.001
Yes, n (%)	101(20.4%)	130(38.9%)	
Maximum lesion diameter			
≤ 1 cm, n (%)	408(82.3%)	173(51.8%)	< 0.001
> 1 cm, n (%)	88(17.7%)	161(48.2%)	
Invaded capsule			
No, n (%)	335(67.5%)	147(44.0%)	< 0.001
Yes, n (%)	161(32.5%)	187(56.0%)	
T stage			
T1-T2, n (%)	475(95.8%)	289(86.5%)	< 0.001
T3-T4, n (%)	21(4.2%)	45(13.5%)	

PTC: papillary thyroid carcinoma; CTC: circulating tumor cell; FU: folate receptor unit

### Comparison of clinicopathological features among PTC patients with or without lateral cervical lymph node metastasis

There were 727 (727/830, 87.6%) PTC patients without and 103 (103/830, 12.4%) PTC patients with lateral cervical lymph node metastasis. Compared with patients without lateral cervical lymph node metastasis, PTC patients with lateral cervical lymph node metastasis had a higher proportion of male patients (31.1% vs. 16.5%) (*p* = 0.001), and patients with < 55 years of age (86.4% vs. 77.4%) (*p =* 0.040). The proportion of multifocal (54.4% vs. 24.1%) (*p* < 0.001), maximum lesion diameter > 1 cm (72.8% vs. 23.9%) (*p* < 0.001), invaded capsule (72.8% vs. 37.6%) (*p* < 0.001), and T3-T4 stage (27.2% vs. 5.2%) (*p* < 0.001) in PTC patients with lateral cervical lymph node metastasis was higher than that in PTC patients without lateral cervical lymph node metastasis, respectively. There were no statistically significant differences in proportion of Hashimoto’s thyroiditis, and preoperative CTCs between these two groups (Table 3).

**Table 3:** Comparison of clinicopathological features among PTC patients with or without lateral cervical lymph node metastasis

Clinicopathological features	Lateral cervical lymph node metastasis		*p* values
	No (*n* = 727)	Yes (*n* = 103)	
Gender			
Male, n (%)	120(16.5%)	32(31.1%)	0.001
Female, n (%)	607(83.5%)	71(68.9%)	
Age (Years)			
< 55, n (%)	563(77.4%)	89(86.4%)	0.040
≥ 55, n (%)	164(22.6%)	14(13.6%)	
Hashimoto’s thyroiditis			
No, n (%)	542(74.6%)	75(72.8%)	0.718
Yes, n (%)	185(25.4%)	28(27.2%)	
Preoperative circulating tumor cells (CTCs) (FU/3mL)			
< 8.7	269(37.0%)	42(40.8%)	0.514
≥ 8.7	458(63.0%)	61(59.2%)	
Multifocal			
No, n (%)	552(75.9%)	47(45.6%)	< 0.001
Yes, n (%)	175(24.1%)	56(54.4%)	
Maximum lesion diameter			
≤ 1 cm, n (%)	553(76.1%)	28(27.2%)	< 0.001
> 1 cm, n (%)	174(23.9%)	75(72.8%)	
Invaded capsule			
No, n (%)	454(62.4%)	28(27.2%)	< 0.001
Yes, n (%)	273(37.6%)	75(72.8%)	
T stage			
T1-T2, n (%)	689(94.8%)	75(72.8%)	< 0.001
T3-T4, n (%)	38(5.2%)	28(27.2%)	

PTC: papillary thyroid carcinoma; CTC: circulating tumor cell; FU: folate receptor unit

### Logistic regression analysis of risk factors of central lymph node metastasis and lateral cervical lymph node metastasis

The results of univariate analysis showed that male (odds ratio (OR): 1.738, 95% confidence interval (CI): 1.220–2.475, *p* = 0.002), age < 55 years old (OR: 1.673, 95% CI: 1.174–2.383, *p* = 0.004), multifocal (OR: 2.492, 95% CI: 1.828–3.398, *p* < 0.001), maximum lesion diameter > 1 cm (OR: 4.315, 95% CI: 3.149–5.911, *p* < 0.001), T3-T4 stage (OR: 3.522, 95% CI: 2.056–6.033, *p* < 0.001), and invaded capsule (OR: 2.647, 95% CI: 1.988–3.525, *p* < 0.001) were significantly associated with central lymph node metastasis. Multivariate regression logistic analysis showed that male (OR: 1.702, 95% CI: 1.151–2.516, *p* = 0.008), age < 55 years old (OR: 1.729, 95% CI: 1.174–2.545, *p* = 0.006), multifocal (OR: 1.921, 95% CI: 1.369–2.695, *p* < 0.001), maximum lesion diameter > 1 cm (OR: 3.251, 95% CI: 2.300-4.593, *p* < 0.001), and invaded capsule (OR: 1.753, 95% CI: 1.268–2.423, *p* = 0.001) were independent risk factors for central lymph node metastasis (Table 4).

**Table 4:** Logistic regression analysis of risk factors of central lymph node metastasis and lateral cervical lymph node metastasis

	Central lymph node metastasis				Lateral cervical lymph node metastasis			
Variables	Univariate		Multivariate		Univariate		Multivariate	
	OR (95% CI)	*p* values	OR (95% CI)	*p* values	OR (95% CI)	*p* values	OR (95% CI)	*p* values
Gender (Male/Female)	1.738 (1.220–2.475)	0.002	1.702 (1.151–2.516)	0.008	2.280 (1.438–3.615)	< 0.001	2.196 (1.279–3.769)	0.004
Age (< 55/≥55, years old)	1.673 (1.174–2.383)	0.004	1.729 (1.174–2.545)	0.006	1.852 (1.027–3.340)	0.041	2.057 (1.062–3.988)	0.033
Hashimoto’s thyroiditis (Yes/No)	1.119 (0.815–1.534)	0.487	1.011 (0.711–1.437)	0.951	1.094 (0.687–1.741)	0.706	0.848 (0.494–1.458)	0.552
Preoperative CTCs (≥ 8.7/<8.7, FU/3mL)	0.982 (0.737–1.308)	0.901	0.982 (0.718–1.343)	0.910	0.853 (0.560–1.299)	0.459	0.771 (0.475–1.250)	0.291
Multifocal (Yes/No)	2.492 (1.828–3.398)	< 0.001	1.921 (1.369–2.695)	< 0.001	3.758 (2.461–5.739)	< 0.001	2.759 (1.708–4.458)	< 0.001
Maximum lesion diameter (> 1 cm/≤1 cm)	4.315 (3.149–5.911)	< 0.001	3.251 (2.300-4.593)	< 0.001	8.513 (5.340–13.570)	< 0.001	5.408 (3.233–9.046)	< 0.001
T stage (T3-T4/T1-T2)	3.522 (2.056–6.033)	< 0.001	1.372 (0.739–2.548)	0.317	6.769 (3.932–11.653)	< 0.001	2.396 (1.241–4.626)	0.009
Invaded capsule (Yes/No)	2.647 (1.988–3.525)	< 0.001	1.753 (1.268–2.423)	0.001	4.454 (2.814–7.051)	< 0.001	2.051 (1.208–3.480)	0.008

PTC: papillary thyroid carcinoma; CTC: circulating tumor cell; FU: folate receptor unit; OR: odds ratio; CI: confidence interval

The results of univariate analysis showed that male (OR: 2.280, 95% CI: 1.438–3.615, *p* < 0.001), age < 55 years old (OR: 1.852, 95% CI: 1.027–3.340, *p* = 0.041), multifocal (OR: 3.758, 95% CI: 2.461–5.739, *p* < 0.001), maximum lesion diameter > 1 cm (OR: 8.513, 95% CI: 5.340–13.570, *p* < 0.001), T3-T4 stage (OR: 6.769, 95% CI: 3.932–11.653, *p* < 0.001), and invaded capsule (OR: 4.454, 95% CI: 2.814–7.051, *p* < 0.001) were significantly associated with lateral cervical lymph node metastasis. Multivariate regression logistic analysis showed that male (OR: 2.196, 95% CI: 1.279–3.769, *p* = 0.004), age < 55 years old (OR: 2.057, 95% CI: 1.062–3.988, *p* = 0.033), multifocal (OR: 2.759, 95% CI: 1.708–4.458, *p* < 0.001), maximum lesion diameter > 1 cm (OR: 5.408, 95% CI: 3.233–9.046, *p* < 0.001), T3-T4 stage (OR: 2.396, 95% CI: 1.241–4.626, *p* = 0.009), and invaded capsule (OR: 2.051, 95% CI: 1.208–3.480, *p* = 0.008) were independent risk factors for lateral cervical lymph node metastasis (Table 4).

## Discussion

The highest proportion of cancers of the endocrine system is thyroid cancer, and in recent decades, the incidence of thyroid cancer has increased at the fastest rate among all malignant tumors in the world [5, 27]. For most PTC patients, the 10-year overall survival rate after surgical resection, radioiodine therapy, and endocrine suppression therapy was 94.8% [28]. The malignant degree of PTC is low and the prognosis is good, but the cervical lymph node metastasis is easy to occur [29], and it is closely related to the increased risk of local recurrence [30]. Sapuppo et al. found that the presence of lateral cervical lymph node metastasis was an important factor for postoperative recurrence and distant metastasis [31]. Currently, total thyroidectomy has been recognized as the most effective treatment for thyroid cancer, but the optimal scope of lymph node dissection remains controversial [16, 32]. Comprehensive regional lymph node dissection in the central or lateral cervical region can improve survival and reduce recurrence rate. Prophylactic lymph node dissection informs the decision of radioactive iodine in PTC [33], and the clinical efficacy of radioactive iodine in the treatment of PTC patients after prophylactic lymph node dissection remains unclear [34]. However, excessive lymph node dissection is associated with a higher incidence of transient or permanent hypoparathyroidism and an increased risk of recurrent laryngeal nerve injury leading to transient or permanent vocal cord paralysis [35–37]. Therefore, preoperative prediction and evaluation of suspected metastatic lymph nodes in the lateral cervical region can provide evidence for clinical decision-making.

In this study, male, age < 55 years old, multifocal, maximum lesion diameter > 1 cm, T3-T4 stage, and invaded capsule were independent risk factors for lateral cervical lymph node metastasis in PTC. Although the majority of PTC patients are young and middle-aged women [38–40], many studies have shown that men are a risk factor for lymph node metastasis in PTC patients. Feng et al. foundthat males were a risk factor for lateral cervical lymph node metastasis in papillary thyroid microcarcinoma (PTMC) patients in a retrospective analysis of 1106 patients [41]. Mao et al. found that the prevalence of lymph node metastasis in male PTC patients was significantly higher than that in female PTC patients [42]. Sapuppo et al. showed that the proportion of male PTC patients with N1b was twice that of male patients with N1a [31]. This study is consistent with the results of previous studies, so more attention should be paid to lymph node metastasis status in male patients with PTC, so as to avoid a relatively poor prognosis due to neglect of lateral cervical lymph node dissection.

The study of Zhang et al. showed that elderly PTC patients were less likely to develop lymph node metastasis [43]. The prospective study performed by Yan et al. also showed that PTC patients with lateral cervical lymph node metastasis were significantly younger than those without lateral cervical lymph node metastasis [44]. A retrospective study of 1033 patients included by Wang et al. pointed out that patients ≤ 30 years old had a higher risk of lateral cervical lymph node metastasis [45]. Mao et al. showed that age < 45 years old is associated with an increased risk of lymph node metastasis in PTC patients [42]. Sapuppo et al. also proposed that PTC patients with N1a and N1b were younger than those with N0 [31]. In this study, the patients were divided into < 55 years old and ≥ 55 years old, age < 55 years old was independent risk factors for lateral cervical lymph node metastasis in PTC, which is basically consistent with the results of previous studies.

According to previous studies, Mao et al. investigated 8 included studies and found that tumor size ≥ 1.0 cm had significantly higher lymph node metastasis rate than < 1.0 cm [42]. Wang et al. included 1033 patients in a retrospective study and concluded that tumor size exceeding 1 cm was an independent risk of lateral cervical lymph node metastasis [45]. A retrospective analysis by Heng et al. showed that the tumor size of lateral cervical lymph node metastasis positive patients was significantly larger than that of the non-lateral cervical lymph node metastasis group [46]. Qubain et al. also concluded that the increase in tumor size was significantly correlated with the increase in the incidence of lateral cervical lymph node metastasis [47]. The results of this study show that the maximum tumor diameter > 1 cm is an independent risk factor for lateral cervical lymph node metastasis in PTC patients, which is consistent with the above results, that is, the risk of lateral cervical lymph node metastasis increases with the increase of tumor volume.

A prospective study by Yan et al. showed that patients with extra thyroidal extension were more likely to develop lateral cervical lymph node metastasis than those without extra-thyroid invasion [44]. An analysis by Mao et al. [42], which included five studies, showed that membrane invasion showed a relatively high lymph node metastasis odds ratio in patients with PTC. Liu et al. compared the clinicopathological features of lateral cervical lymph node metastasis patients with and without lateral cervical lymph node metastasis, and compared with the lateral cervical lymph node metastasis negative group, the lateral cervical lymph node metastasis positive group had more features of capsule invasion [48]. In addition, there are few studies on circulating tumor cells in lymph node metastasis of PTC patients. A prospective study showed that CTCs had high diagnostic efficacy in patients with suspected thyroid nodules [49]. Wang et al. found that patients with high CTCs level have shorter progression-free survival (PFS) [50]. In this study, preoperative CTCs ≥ 8.7 FU/3mL was not risk factor for lateral cervical lymph node metastasis in PTC.

This study provides valuable reference data for the risk assessment of lateral cervical lymph node metastasis in patients with PTC. Of course, this study still has the following limitations. This study is a single-center retrospective study, and the existence of multiple types of bias such as selection bias cannot be ruled out. In addition, due to the limited number of cases that can be collected, the included sample size is limited, and the research results may be slightly biased. Therefore, the conclusions of this study need to be further verified by prospective studies involving more patients in multiple centers to obtain more accurate and rigorous results.

## Conclusions

In summary, male, age < 55 years old, multifocal, maximum lesion diameter > 1 cm, T3-T4 stage, and invaded capsule were independent risk factors for lateral cervical lymph node metastasis in PTC. We believe that comprehensive consideration of these indicators can predict whether there is lateral cervical lymph node metastasis in PTC patients, and provide reference data for the selection of lymph node dissection scope in clinical surgery.

## Data Availability

No datasets were generated or analysed during the current study.
